# Supplementary material for: A variety of soliton solutions of time M-fractional: Non-linear models via a unified technique
Source: PLoS One. 2024 Apr 26;19(4):e0300321. doi: 10.1371/journal.pone.0300321 (PMC11051631; doi:10.1371/journal.pone.0300321)
Supplement: S1 File — (DOCX) [file pone.0300321.s001.docx]

For plotting diverse phenomena, we used different simulation data of free variable.

| Simulation data | Name of wave pattern |
| --- | --- |
| $\alpha=d=0.5=-\gamma,h=5,\mathcal{l=-}4,p=1$ and $[\lambda=0.3, 0.6,0.9]$. | Figure 01: kink shape soliton solution of eq. (13) |
| $d=0.5=\gamma,\alpha=0.167,h=5,\mathcal{l=-}4,p=1$ and $[\lambda=0.3, 0.6,0.9]$ | Figure 02: collision of anti-kink and lump wave of eq. (15) |
| $\gamma=0.1,d=0.5,h=1,\alpha=-0.25,\mathcal{l=}0.5,p=1$ and $[\lambda=0.3, 0.6,0.9]$ | Figure 03: lump wave solution of eq. (15) |
| $\alpha=-0.167,d=0.5,\gamma=0.5,h=5,\mathcal{l=-}4,p=1$ and $[\lambda=0.3, 0.6,0.9]$ | Figure 04: lump wave solution of eq. (17) |
| $\gamma=-0.5,\alpha=1,h=-1,\mathcal{l=}0.2,d=0.5,p=-1$ and $[\lambda=0.3, 0.6,0.9]$ | Figure 05: collision of kink and lump wave of eq. (19) |
| $\gamma=-0.5,\alpha=0.5,h=0.25,d=0.5,p=-1$ and $[\lambda=0.3, 0.6,0.9]$ | Figure 06: soliton solution of eq. (19) |
| $\gamma=0.1,\alpha=0.5,h=4,\mathcal{l=}0.5,d=0.5,p=1$ and $[\lambda=0.3, 0.6,0.9]$ | Figure 07: collision of kink and lump wave of eq. (23) |
| $\gamma=0.1,\alpha=0.5,h=4,\mathcal{l=}0.5,d=1,p=1$ and $[\lambda=0.3, 0.6,0.9]$ | Figure 08: interaction of lump and bell type soliton solution of eq. (23) |
| $\gamma=-0.5,\alpha=0.5,h=4,\mathcal{l=-}4,d=0.5,p=1$ and $[\lambda=0.3, 0.6,0.9]$ | Figure 09: Bright bell shape solution of eq. (23) |
| $d=0.5=\alpha=-\gamma,h=5,\mathcal{l}=-4,p=1$ and $[\lambda=0.3, 0.6,0.9]$ | Figure 10: dark bell shape solution of eq. (23) |
| $p=1,\mathcal{l=}0.5,h=-0.75, \gamma=-0.5, k=-0.25, a=1,d=0.5,b=0.5$ and $[\lambda=0.3, 0.6,0.9]$ | Figure 11: periodic lump wave solution of eq. (30) |
| $d=0.5,h=1,p=1,\mathcal{l=}0.5,\gamma=0.20, k=1, a=0.1,b=-0.33$ and $[\lambda=0.3, 0.6,0.9]$ | Figure 12: periodic soliton solution of eq. (34) |
| $d=0.5,h=1,p=1,\mathcal{l=}0.5,\gamma=0.20, k=1, a=0.25,b=0.33$ and $[\lambda=0.3, 0.6,0.9]$ | Figure 13: periodic wave solution of eq. (36) |
| $d=0.5,h=-0.75,p=1,\mathcal{l=}0.5,\gamma=-0.5, k=-1, a=0.5,b=0.5$ and $[\lambda=0.3, 0.6,0.9]$ | Figure 14: soliton solution of eq. (38) |
| $d=1,h=0.5,p=1,\mathcal{l=}0.5,\gamma=-0.50, k=2, a=1.5,b=1$ and $[\lambda=0.3, 0.6,0.9]$ | Figure 15: kink soliton solution of eq. (40) |
| $d=0.5,h=0.75,p=1,\mathcal{l=}0.5,\gamma=0.50, k=-1, a=0.5,b=0.5$ and $[\lambda=0.3, 0.6,0.9]$ | Figure 16: periodic soliton solution of eq. (45) |
