# Supplementary material for: A variety of soliton solutions of time M-fractional: Non-linear models via a unified technique
Source: PLoS One. 2024 Apr 26;19(4):e0300321. doi: 10.1371/journal.pone.0300321 (PMC11051631; doi:10.1371/journal.pone.0300321)
Supplement: Attachment — Submitted filename: Reviewer Response.docx [file pone.0300321.s002.docx]

**Reviewer Response:**

5. Review Comments to the Author

Please use the space provided to explain your answers to the questions above. You may also include additional comments for the author, including concerns about dual publication, research ethics, or publication ethics. (Please upload your review as an attachment if it exceeds 20,000 characters)

**Reviewer #1**: I carefully read the paper and have identified several issues needing improvement regarding its written expression, particularly concerning numerous errors in English language usage. Additionally, the paper could benefit from improved organization.

**Ans.: We have fixed the errors checking overall manuscript with help of Grammarly software.**

The paper's solutions need to be more novelty and establish a comparative analysis with the solutions proposed by other authors.

**Ans. We explored novel M-fractional form of two models. Compared the solutions with various fractional derivatives and even included a comparison section “6. Comparisons and novelty of this manuscript”.**

What is the underlying motivation behind the research presented in this paper?

**Ans. We explored novel M-fractional form of two models and compared the solutions with various fractional derivatives and even included a comparison section “6. Comparisons and novelty of this manuscript”. Also see red part in the introduction section.**

In the absence of novel solutions, the author has the option to introduce an additional fractional derivative, such as a beta-derivative or conformable fractional derivative.

**Ans. The beta-derivative or conformable fractional derivative are old but we explored here the truncated M-frational derivative and compared three fractional derivatives in each 2D graphs.**

Based on my evaluation, I cannot endorse this paper's publication, as indicated in my concluding remarks.

**Ans. We novelty, comparison and effects of various fractional derivatives are shown. See all 2-D graphs and section 6. Thank you for help in improvement the manuscript.**

Reviewer #2: Please consider the review report.

My evaluation on Ms. No.: PONE-D-23-16176 in PLOS ONE Journal

**My evaluation on Ms. No.: PONE-D-23-16176 in Plos One Journal:**

The paper is interesting, and the results are useful for the wide readership. The authors must look carefully to the below points:

1. The whole manuscript should be checked for typos and grammatical errors. There are various types of errors in the manuscript. An overall review is needed for fixing the grammatical and typos errors in the manuscript.

**Ans.: We have fixed the errors checking overall manuscript and the plagiarism reduces to 8%.**

2. The Abstract should contain answers to the following questions: What problem was studied and why is it important? What methods were used? What are the important results? What conclusions can be drawn from the results? What is the novelty of the work and where does it go beyond previous efforts in literature?

**Ans.: We rewrite the abstract according to your suggestion which fixed all your quaternary.**

3. The keywords must not contain any word that existed in the title.

**Ans.: Modified it.**

4. Abstract should not include any abbreviations.

**Ans.: We modified the abbreviations from abstract**.

5. A professional proof-reading is required for the whole manuscript.

Ans.: **We have fixed the errors checking overall manuscript by expert (Alrazi Abdejabber).**

6. The authors should explain the limitations of this work in the introduction section.

**Ans.:** **M-fractional form of these models was not explored in the previous literature was included in the introduction section. (See red color portions). The limitation is that no muti-soliton found in this fractional model.**

7. The authors should explain why the study is useful with a clear statement of novelty or originality by providing relevant information in the introduction and conclusion sections.

**Ans.: We explored more in the introduction and conclusions by novelty. (See red color portions).**

8. The author should add some more discussions on figures and numerical simulation in the conclusion and introduction section.

**Ans.: We added some explanation in conclusion and introduction section**

9. References [1-6] and [27-34] must be written in detail.

**Ans.: All references are written in detail.**

10. References [30] and [31] are missing and they must be added before reference [32].

**Ans.: All References are explained in introduction sector**.

11. Looking through the manuscript, the author should present the physical motivation for the nonlinear waves for Eq. (1) and (2). Why did the author consider these equations?

**Ans.: The important of the models in the introduction section are included properly. (See red color portions).**

12. The introduction needs to be improved by the recent developments in the field of the soliton theory as well as its applications. For this purpose, the authors can add the following references to enrich the

introductory section: Results in Physics, 2021, 23, 104043; Physica Scripta, 2021, 96(9), 094006; Results in Physics, 2020, 19, 103329; Advances in Difference Equations, 2020, 2020(1), 494; Communications in Theoretical Physics, 2021, 37(10), 105007; Results in Physics, 2023, 49, 106537; European Journal Physical Plus, 2023, 138(6),502; Alxandria Engineering Journal, 2023, 74, 585–597; Fractal and Fractional, 2023, 7(6), 426; Nonlinear Dynamics, 2023, 111, 9457-9466; Optik, 2023, 281, 170817; and Communications in Theoretical Physics, 2023.

<https://iopscience.iop.org/article/10.1088/1572-9494/ace155/meta>

**Ans.: We added some article in [19, 20, 21, 22] and [35, 36].**

13. The conclusion section is too short. The authors should comment on figures in detail in the conclusion section. The conclusion section must also contain new findings of the paper.

**Ans.: We rewrite the conclusion sector.**

14. The authors should provide the future scope of the work in the conclusion section.

**Ans.: We added the future scope in conclusion.**

15. Correct Reference to References.

**Ans.: We rewrite it and corrected all references in a unique form**.

After the above revision, I recommended this paper for publication in your journal
