# Supplementary material for: A variety of soliton solutions of time M-fractional: Non-linear models via a unified technique
Source: PLoS One. 2024 Apr 26;19(4):e0300321. doi: 10.1371/journal.pone.0300321 (PMC11051631; doi:10.1371/journal.pone.0300321)
Supplement: Attachment — Submitted filename: Reviewer Response.docx [file pone.0300321.s003.docx]

Journal requirements:

When submitting your revision, we need you to address these additional requirements.

1. Please ensure that your manuscript meets PLOS ONE's style requirements, including those for file naming. The PLOS ONE style templates can be found at

<https://journals.plos.org/plosone/s/file?id=wjVg/PLOSOne_formatting_sample_main_body.pdf> and

<https://journals.plos.org/plosone/s/file?id=ba62/PLOSOne_formatting_sample_title_authors_affiliations.pdf>

**Ans.: We done the necessary change to be the plos one journal style such as title, references, and figure number.**

2. Please note that PLOS ONE has specific guidelines on code sharing for submissions in which author-generated code underpins the findings in the manuscript. In these cases, all author-generated code must be made available without restrictions upon publication of the work. Please review our guidelines at <https://journals.plos.org/plosone/s/materials-and-software-sharing#loc-sharing-code> and ensure that your code is shared in a way that follows best practice and facilitates reproducibility and reuse.

**Ans.: we are agreed with the PLOS ONE the specific guidelines on code sharing process.**

3. Thank you for stating the following financial disclosure:

“Include this sentence at the end of your statement: The funders had no role in study design, data collection and analysis, decision to publish, or preparation of the manuscript.”

At this time, please address the following queries:

a) Please clarify the sources of funding (financial or material support) for your study. List the grants or organizations that supported your study, including funding received from your institution.

**Ans.: There are no sources of funding (financial or material support) for this study or grants or organizations that supported this study, including funding received from your institution.**

b) State what role the funders took in the study. If the funders had no role in your study, please state: “The funders had no role in study design, data collection and analysis, decision to publish, or preparation of the manuscript.”

**Ans.: The funders had no role in study design, data collection and analysis, decision to publish, or preparation of the manuscript.**

c) If any authors received a salary from any of your funders, please state which authors and which funders

**Ans.: There are no salary that received from any of your funders.**

d) If you did not receive any funding for this study, please state: “The authors received no specific funding for this work.”

Please include your amended statements within your cover letter; we will change the online submission form on your behalf.

**Ans.: The authors received no specific funding for this work.**

4. In your Data Availability statement, you have not specified where the minimal data set underlying the results described in your manuscript can be found. PLOS defines a study's minimal data set as the underlying data used to reach the conclusions drawn in the manuscript and any additional data required to replicate the reported study findings in their entirety. All PLOS journals require that the minimal data set be made fully available. For more information about our data policy, please see <http://journals.plos.org/plosone/s/data-availability>.

**Ans.: I am sorry to inform you that we do not use any collected data in this manuscript. So we have no data. For plotting diverse phenomena, we used different simulation data of free variable. The data are uploaded as the supporting file.**

Upon re-submitting your revised manuscript, please upload your study’s minimal underlying data set as either Supporting Information files or to a stable, public repository and include the relevant URLs, DOIs, or accession numbers within your revised cover letter. For a list of acceptable repositories, please see <http://journals.plos.org/plosone/s/data-availability#loc-recommended-repositories>. Any potentially identifying patient information must be fully anonymized.

Important: If there are ethical or legal restrictions to sharing your data publicly, please explain these restrictions in detail. Please see our guidelines for more information on what we consider unacceptable restrictions to publicly sharing data: <http://journals.plos.org/plosone/s/data-availability#loc-unacceptable-data-access-restrictions>. Note that it is not acceptable for the authors to be the sole named individuals responsible for ensuring data access.

We will update your Data Availability statement to reflect the information you provide in your cover letter.

5. PLOS requires an ORCID iD for the corresponding author in Editorial Manager on papers submitted after December 6th, 2016. Please ensure that you have an ORCID iD and that it is validated in Editorial Manager. To do this, go to ‘Update my Information’ (in the upper left-hand corner of the main menu), and click on the Fetch/Validate link next to the ORCID field. This will take you to the ORCID site and allow you to create a new iD or authenticate a pre-existing iD in Editorial Manager. Please see the following video for instructions on linking an ORCID iD to your Editorial Manager account: <https://www.youtube.com/watch?v=_xcclfuvtxQ>

**Ans.: we added the ORCID iD for the corresponding author.**

Additional Editor Comments:

Revise.


[Note: HTML markup is below. Please do not edit.]

Reviewers' comments:

**Reviewer's Responses to Questions**

**Comments to the Author**

1. Is the manuscript technically sound, and do the data support the conclusions?

The manuscript must describe a technically sound piece of scientific research with data that supports the conclusions. Experiments must have been conducted rigorously, with appropriate controls, replication, and sample sizes. The conclusions must be drawn appropriately based on the data presented.

Reviewer #1: Yes

Reviewer #2: Yes

2. Has the statistical analysis been performed appropriately and rigorously?

Reviewer #1: N/A

Reviewer #2: Yes

3. Have the authors made all data underlying the findings in their manuscript fully available?

The [PLOS Data policy](http://www.plosone.org/static/policies.action#sharing) requires authors to make all data underlying the findings described in their manuscript fully available without restriction, with rare exception (please refer to the Data Availability Statement in the manuscript PDF file). The data should be provided as part of the manuscript or its supporting information, or deposited to a public repository. For example, in addition to summary statistics, the data points behind means, medians and variance measures should be available. If there are restrictions on publicly sharing data—e.g. participant privacy or use of data from a third party—those must be specified.

Reviewer #1: No

Reviewer #2: Yes

4. Is the manuscript presented in an intelligible fashion and written in standard English?

PLOS ONE does not copyedit accepted manuscripts, so the language in submitted articles must be clear, correct, and unambiguous. Any typographical or grammatical errors should be corrected at revision, so please note any specific errors here.

Reviewer #1: Yes

Reviewer #2: Yes

5. Review Comments to the Author

Please use the space provided to explain your answers to the questions above. You may also include additional comments for the author, including concerns about dual publication, research ethics, or publication ethics. (Please upload your review as an attachment if it exceeds 20,000 characters)

**Reviewer #1:**

1. Remove all typos from the text.

**Ans.: Necessary correction has been done in the revised manuscript.**
2. The introduction section should be improved by incorporating similar recent works.

**Ans.: We added some recent article in introduction section. If you have any important and related article then please send me the doi number.**
3. Authors should highlight some applications of this study.

**Ans.: We highlighted some applications of this study in section 1 and 5.**
4. What is the novelty of this research work?
**Ans.: In comparison section, we show the novelty of this article.**

5. The explanation of obtained results should be backed by fundamental knowledge and

Principles.
**Ans.: The obtained solutions are explored with its importance in section 5.1 and 5.2.**

6. The English language should be well checked.

**Ans.: English language has been checked properly.**

Reviewer #2: Each of the issues raised by the reviewers was addressed by the authors. The article was revised based on the reviewers' comments and errors were corrected. I express my opinion that it is appropriate for the article to be accepted for publication in present form.

**Ans.: Thanks for your recommendation.**
